# Exploring *Mycoplasma ovipneumoniae* NXNK2203 infection in sheep: insights from histopathology and whole genome sequencing

**DOI:** 10.1186/s12917-023-03866-z

**Published:** 2024-01-10

**Authors:** Jiandong Wang, Hongyan Liu, Abdul Raheem, Qing Ma, Xiaojun Liang, Yanan Guo, Doukun Lu

**Affiliations:** 1 NingXia Academy of Agricultural and Forestry Sciences, Yinchuan, 750002 China; 2 School of Agriculture, Ningxia University, Yinchuan, 750021 China; 3 National Key Laboratory of Agricultural Microbiology, Huazhong Agricultural University, Wuhan, 430070 China

**Keywords:** *Mycoplasma ovipneumoniae*, Sheep, Respiratory disease, Histopathology, Whole genome sequencing

## Abstract

**Background:**

*Mycoplasma ovipneumoniae* (*M. ovipneumoniae*) is a significant pathogen causing respiratory infections in goats and sheep. This study focuses on investigating vulnerability of Hu sheep to *M. ovipneumoniae* infection in the context of late spring’s cold weather conditions through detailed autopsy of a severely affected Hu sheep and whole genome sequencing of *M. ovipneumoniae*.

**Results:**

The autopsy findings of the deceased sheep revealed severe pulmonary damage with concentrated tracheal and lung lesions. Histopathological analysis showed tissue degeneration, mucus accumulation, alveolar septum thickening, and cellular necrosis. Immunohistochemistry analysis indicated that *M. ovipneumoniae* was more in the bronchi compared to the trachea. Genome analysis of *M. ovipneumoniae* identified a 1,014,835 bp with 686 coding sequences, 3 rRNAs, 30 tRNAs, 6 CRISPRs, 11 genomic islands, 4 prophages, 73 virulence factors, and 20 secreted proteins.

**Conclusion:**

This study investigates the vulnerability of Hu sheep to *M. ovipneumoniae* infection during late spring’s cold weather conditions. Autopsy findings showed severe pulmonary injury in affected sheep, and whole genome sequencing identified genetic elements associated with pathogenicity and virulence factors of *M. ovipneumoniae*.

**Supplementary Information:**

The online version contains supplementary material available at 10.1186/s12917-023-03866-z.

## Introduction

Bacterial and viral respiratory diseases pose significant challenges to the health of livestock populations worldwide [1–3]. Respiratory disorders in ovine species often arise as a consequence of immunosuppressive stress conditions, either as secondary infections or as primary infections caused by bacterial and/or viral agents [4]. These respiratory ailments represent prominent health concerns in livestock populations, exerting a substantial impact on animal welfare and inflicting considerable economic burdens [5]. Globally, *M. ovipneumoniae* assumes critical significance as a leading causative agent of respiratory disease in sheep [6, 7].

Mycoplasma species are postulated to have evolved from Gram-positive ancestors [5], characterized by their possession of small genomes [8]. In the natural environment, Mycoplasmas are widely distributed and serve as significant parasites affecting humans, mammals, plants, and other organisms [9]. Among these species, *M. ovipneumoniae* specifically infects sheep and goats. Initially isolated from the lungs of sheep exhibiting adenomatosis in Scotland in 1963, and was formally designated as *M. ovipneumoniae* in 1972 [10]. The clinical manifestations of diseased sheep are cough, runny nose, asthma, progressive emaciation, and pulmonary interstitial proliferative inflammation, often with concealed and persistent infection [11, 12]. In the past few decades, it has spread widely around the world, causing huge economic losses to the sheep industry [13].

*M. ovipneumoniae* has been the subject of scientific inquiry for several decades. Despite extensive research efforts, many aspects of this microorganism remain elusive. The predominant method currently employed for the isolation and identification of *M. ovipneumoniae* is the analysis of the 16S rRNA gene sequence, a technique known as Sanger sequencing. Through this approach, *M. ovipneumoniae* has been classified within the hyponeumoniae group of mycoplasmas, specifically within the neuroyticum/hyoneumoniae cluster, based on the analysis of 16 S rRNA and rpoB gene sequences [14]. However, a comprehensive understanding of the bacterium’s virulence genes necessitates the application of whole-genome sequencing. In 2011, the first complete genome sequence of *M. ovipneumoniae* was reported, derived from a strain designated SC01 that was obtained from a goat [15]. Analysis of the SC01 genome revealed the presence of at least eight coding sequences (CDSs) responsible for encoding proteins similar to adhesin-like proteins found in *Mycoplasma. Hyopneumoniae* (*M. ovipneumoniae*). The identification and characterization of such genes hold significance in understanding the adhesive properties of *M. ovipneumoniae* and their potential implications in pathogenesis.

Nonetheless, the highly repetitive nature of *M. ovipneumoniae* genomes poses challenges when utilizing Illumina short-read sequencing technology. These challenges primarily stem from difficulties in read mapping, which are exacerbated by CG biases induced during library preparation [16]. To overcome these limitations, long-read sequencing technologies have emerged as valuable tools in various scientific domains, including metagenomics and de novo genome assemblies. Leveraging the advancements in sequencing technology, we employed molecular real-time technology (SMRT) in this investigation to perform whole-genome sequencing of *M. ovipneumoniae* strain NXNK2203. This strain was isolated from the lungs of Hu sheep that had succumbed to the infection on a farm in Ningxia.

The comprehensive analysis of the entire genome of *M. ovipneumoniae* serves multiple crucial purposes. Firstly, it enables the exploration of known gene functions, shedding light on the mechanisms underlying the bacterium’s pathogenicity. Secondly, it facilitates the identification of previously undiscovered gene information, potentially uncovering novel virulence factors or therapeutic targets. Finally, this genomic information lays the foundation for the development of vaccines, aiding in the prevention and control of *M. ovipneumoniae* infections in ovine populations.

## Materials and methods

### Necropsy, histopathology and immunohistochemistry

The lesions on the surfaces or cross-sections of lungs and trachea of dead sheep were examined. The gross pathology analysis focused on observing lung color changes, adhesions between the lungs and the chest wall, pleural effusion, and surface lesions of the trachea or lungs. This examination provided a macroscopic overview of any abnormalities or visible pathological features.

Following the gross pathology examination, samples of lung tissue, trachea, and submandibular lymph nodes were taken for further histopathological analysis, which allows detailed observation of the cellular and tissue structures. For this, tissues were subjected to fixation, embedding, sectioning, and staining with hematoxylin and eosin (H&E) using established conventional techniques [17]. Subsequently, the sections were evaluated utilizing light microscopy. For immunohistochemistry, the specimens were prepared using the primary antibody (*M. ovipneumoniae* positive serum) and secondary antibody (rabbit anti-sheep IgG HRP).

### Bacterial isolation and identification

The tubes containing the tissue homogenate and tracheal swabs in phosphate-buffered saline (PBS) were subjected to vortexing, followed by the inoculation of the suspension in pleuropneumonia-like organisms (PPLO) agar plates supplemented with horse serum. The agar plates were then placed in an incubator set at 37 °C for a duration of 24 h allowing for the selection of individual single bacterial colonies. Then PPLO screening medium was inoculated with a single bacterial colony and placed at the same temperature for 24–48 h. The resulting yellowed culture medium was subsequently filtered through a 0.22 μm filter membrane and further inoculated in PPLO medium. After another 48-hour incubation period at 37 °C, colony morphology was observed using a microscope.

The purified and propagated bacterial suspension was collected to extract DNA using the Bacterial DNA Kit D3350 from Omega. For the specific identification of *M. ovipneumoniae*, the Transketolase-F (GTTGGTGGCAAAAGTCACTAG) and Transketolase-R (CTTGACATCACTGTTTCGCTG) primers were utilized for the polymerase chain reaction (PCR) for amplification of the Transketolase gene. The resulting PCR products were subsequently analyzed using 1% agarose gel electrophoresis.

### Whole genome sequencing

To identify and characterize specific genetic elements associated with pathogenicity, antibiotic resistance, and virulence factors of bacteria, the whole genome sequencing was performed as WGS of bacteria plays a crucial role in unraveling the intricate relationship between these microorganisms and the onset of deadly diseases.

The genomic DNA of bacteria was extracted utilizing the aforementioned methodology. The concentration and purity of the DNA were assessed using a Nanodrop ND-2000 spectrophotometer, while the integrity of the DNA was evaluated through agarose gel electrophoresis. Subsequently, the high-quality samples were subjected to PacBio sequencing technology utilizing a single-molecule real-time (SMRT) chip (Biomarker Technologies, Beijing, China). For genome assembly, the Hifiasm software was employed. Additionally, the Circlator v1.5.5 software was utilized to circularize and adjust the starting site of the assembled genome. Furthermore, Pilon v1.22 software was utilized to enhance the accuracy of the second-generation data by performing additional corrections, resulting in a more precise genome sequence.

### Genome annotation and analysis

Putative CDS were identified by Prodigal v2.6.3 software [18]. Repetitive sequences were predicted utilizing RepeatMasker v4.0.5 software [19]. Ribosomal RNA (rRNA) and transfer RNA (tRNA) were predicted employing Infernal v1.1.3 [20] and tRNAscan-SE v2.0 [21] respectively. Clustered Regularly Interspaced Palindromic Repeats (CRISPR) were found by CRT v1.2 [22]. Gene island was predicted using IslandPath-DIMOB v0.2 software [23]. Pre-bacteriophages within bacterial genome were obtained via PhiSpy v2.3 [24].

Promoter regions were obtained from PromPredict v1 software [25]. Functional predictions were based on multiple databases, including the Non-Redundant protein database (Nr) [26], Gene Ontology database (GO) [27], Kyoto Encyclopedia of Genes and Genomes database (KEGG) [28], evolutionary genealogy of genes: Non-supervised Orthologous (eggNOG) database [29], Pfam database [30], Swissprot, and TrEMBL database [31]. Additionally, specific databases were employed for functional annotations, namely the Carbohydrate-active enzymes database (CAZy) [32], Transporter Classification Database (TCDB) [33], Comprehensive Antibiotic Research Database (CARD) [34], and Virulence Factor Database (VFDB) [35]. Transmembrane helices and Signal peptides were searched using TMHMM 2.0 and Signal v4.0 software [36] respectively. Finally, the genome circle was mapped using Circos v0.66 software [37].

### Phylogenetic tree construction (phylogenetic analysis)

In order to comprehend the evolutionary history and interrelationship of organisms, a phylogenetic tree was constructed by leveraging shared traits among 19 strains of *M. ovipneumoniae*. The whole genome data of these strains were taken from the National Center for Biotechnology Information database (additional file 1). Protein FASTA (.faa) files containing comprehensive protein information for each strain were extracted from the assemblies. Through the application of OrthoFinder, single-copy genes were identified. Subsequently, all proteins were concatenated and subjected to sequence alignment using the MUSCLE algorithm. Employing the Maximum-Likelihood method with 1000 nonparametric bootstrap replicates in FastTree, a phylogenetic tree was successfully generated.

## Results

### Histopathological examination

**Fig. 1:**
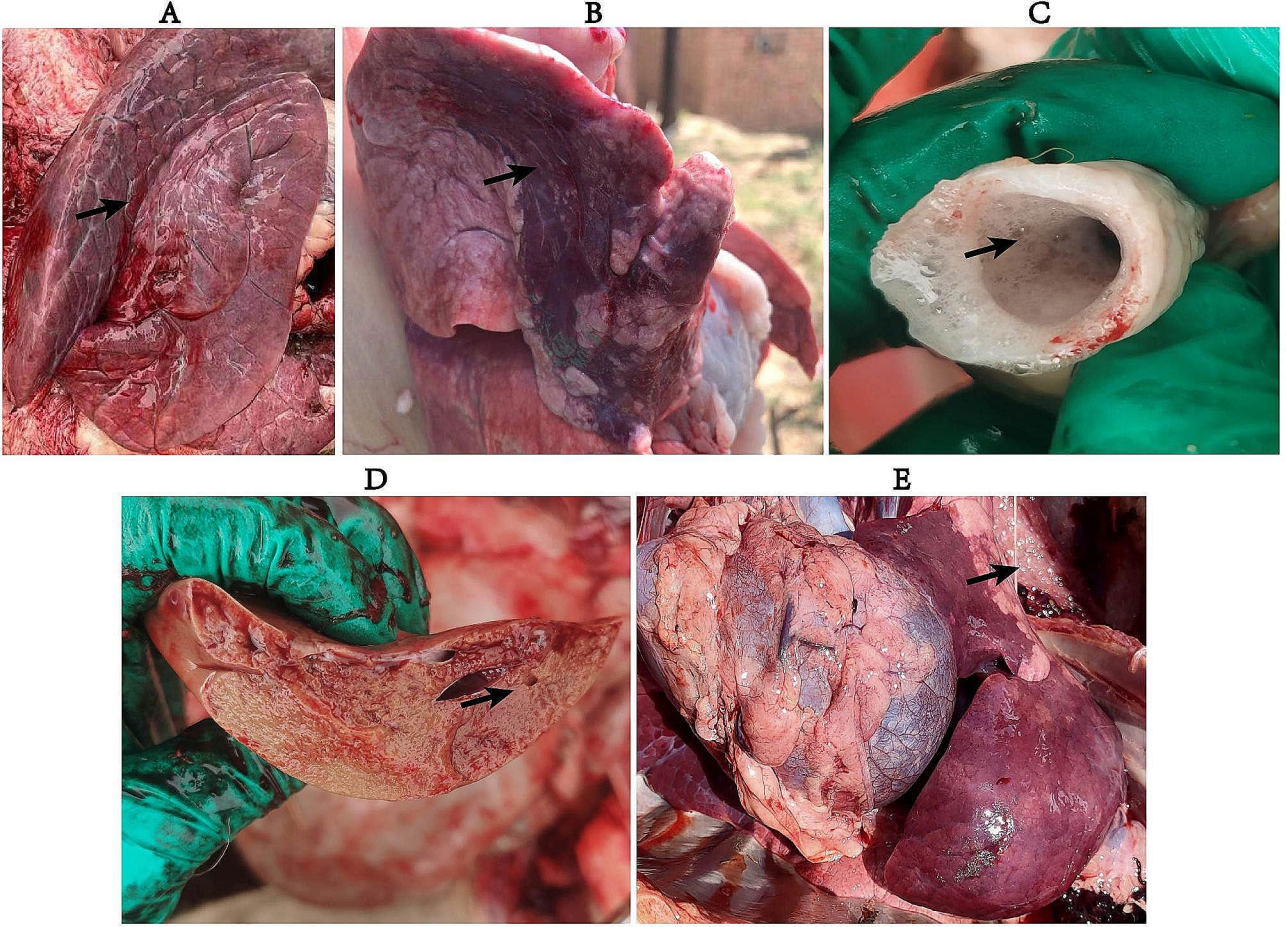
Pathological anatomy of dead sheep. **A**-**B**: upper lobes of lungs; **C**: tracheal mucus; **D**: lung mucus; **E**: adhesion between lung and chest

**Fig. 2:**
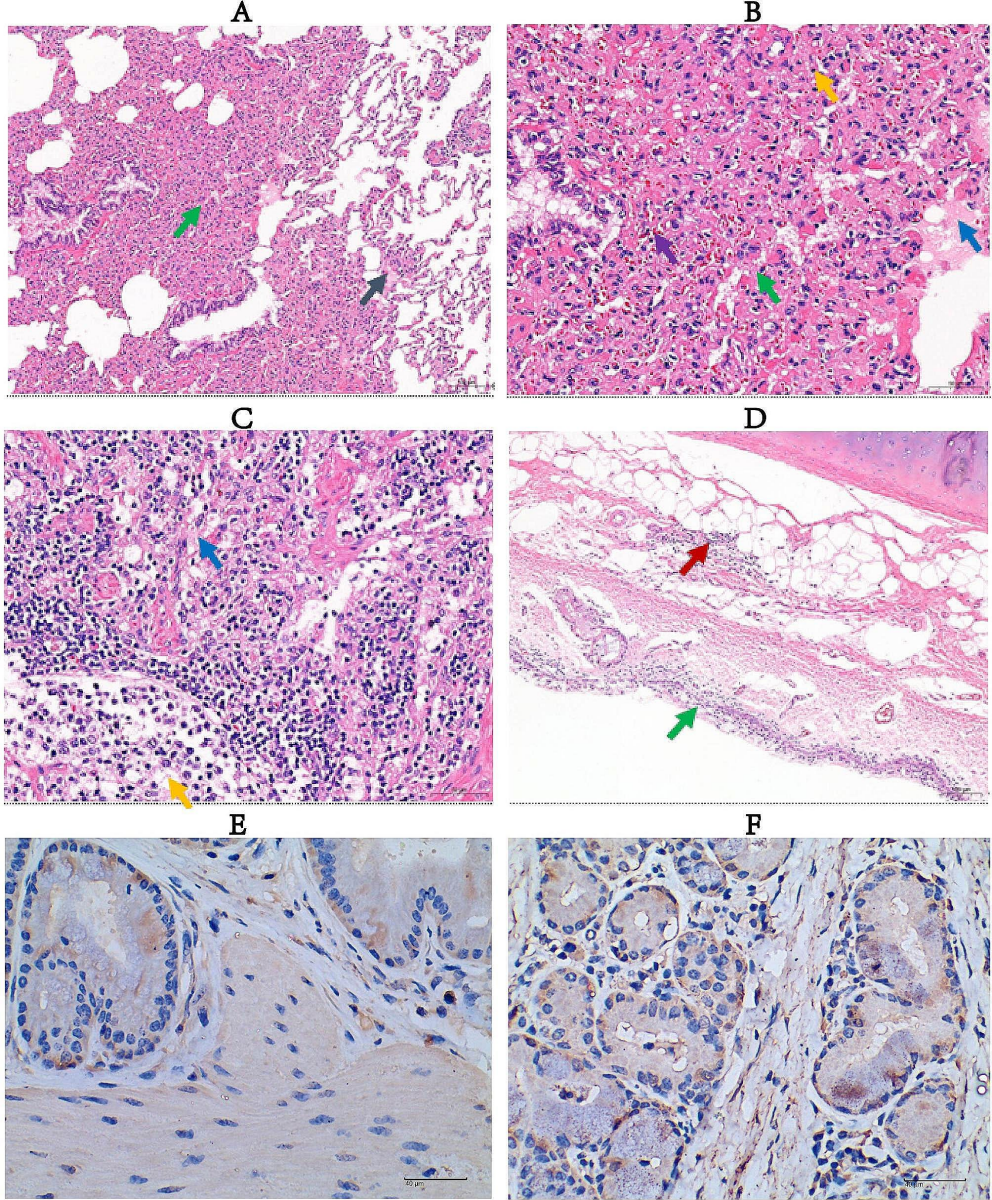
Pathological sections (H&E staining) and immunohistochemical analysis of trachea and bronchus. A: lungs (100 ×), alveolar septum thickening (green arrow), macrophages (black arrow); B: lungs (400 ×), alveolar epithelial cell necrosis (green arrow), fibrinoid exudation (blue arrow), lymphocytes (yellow arrow), telangiectasia (purple arrow); C: submandibular lymph nodes (400 ×), necrotic lymphocytes (yellow arrow), fibrinoid exudation (blue arrow), bronchus (400 ×), mucosal epithelial necrosis and exfoliation (green arrow), inflammatory cell infiltration (red arrow). Immunohistochemistry analysis; D: Trachea (400×), E: Bronchus (400×), the nucleus in the image is displayed blue, and the positive signal of *M. ovipneumoniae* is displayed in brownish yellow

Autopsy of dead sheep showed that the lesions were mainly concentrated in the trachea and lungs. Specifically, there was profound degeneration of solid flesh observed in the upper lobes of lungs (Fig. 1A and B), Moreover, a significant accumulation of mucus was evident in the trachea and lungs (Fig. 1C and D), along with the presence of adhesions between the lungs and the thoracic cavity (Fig. 1E). Furthermore, the histopathological analysis displayed thickening of the alveolar septum, an increase in macrophages, necrosis of pulmonary epithelial cells, fibrinoid exudation, lymphocytosis, and telangiectasia (Fig. 2A and B). Submandibular lymph nodes exhibited enhanced cellular necrosis and fibrinoid exudation (Fig. 2C). Similarly, the bronchi exhibited extensive mucosal necrosis, exfoliation, and inflammatory cell infiltration (Fig. 2D). The confluence of clinical presentations and the above findings unequivocally indicated a grave extent of pulmonary injury in deceased ovine species.

### Immunohistochemistry

Our immunohistochemistry analysis demonstrated a pronounced abundance of *M. ovipneumoniae* within the bronchial regions than trachea as illustrated in Fig. 2E and F. The observed quantities markedly exceeded those identified in the bronchial regions sections, suggesting a preferential localization or heightened susceptibility of these regions to *M. ovipneumoniae* colonization.

### Bacterial isolation and identification

**Fig. 3:**
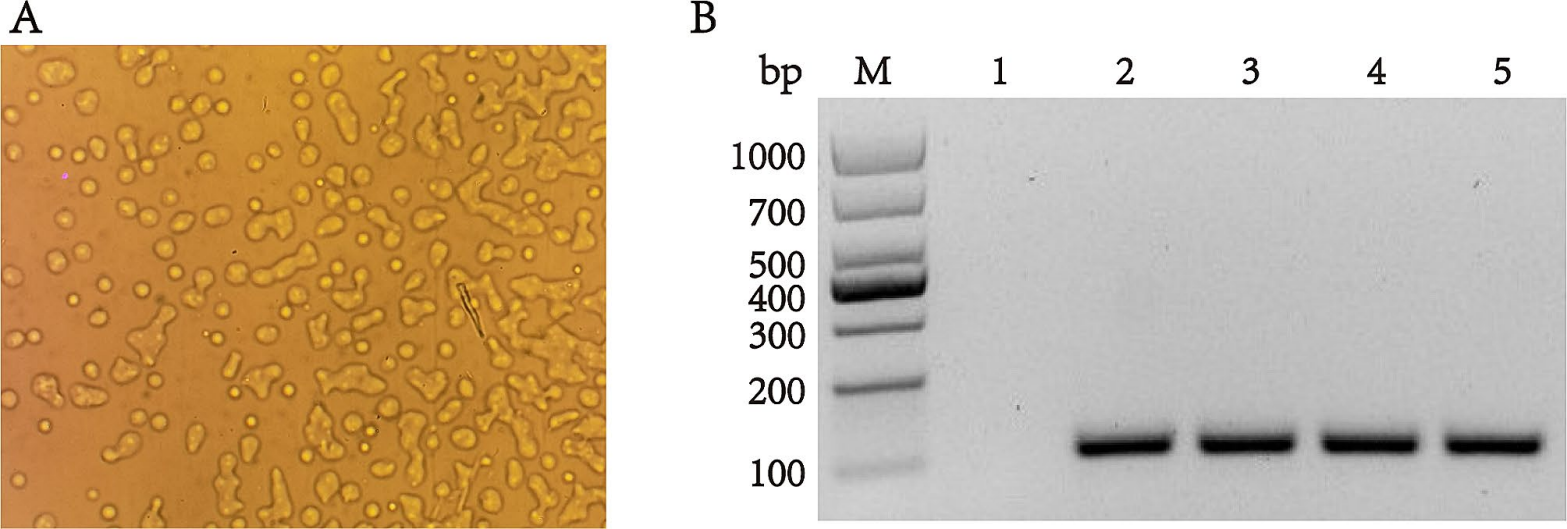
Specific identification of *M. ovipneumoniae***A**: Morphological observation of *M. ovipneumoniae* in PPLO agar medium (10×). Full-length agar growth plat image is presented in additional file 12 (Figure. S2 A); **B**: PCR was used to identify the Transketolase gene specific for *M. ovipneumoniae*. M: Marker; 1: Negative control; 2–5: Clinical isolate strain of *M. ovipneumoniae* NXNK2203. Full-length picture of PCR gel are presented in additional file 12 (Figure. S2 B)

To investigate the etiology behind the mortality observed in Hu sheep, the bacterial solution was isolated and subjected to identification procedures. The outcomes revealed the presence of a distinctive colony morphology resembling a “fried egg” on the PPLO agar plate (Fig. 3A and Fig. S2A), a characteristic commonly associated with mycoplasma species. To further ascertain the causative agent, we employed specific primers targeting the Transketolase gene of *M. ovipneumoniae* for amplification, which resulted in the generation of positive bands across all analyzed samples (Fig. 3B and Fig. S2B). Consequently, we attributed the mortality to a novel strain derived from deceased Hu sheep and designated it as *M. ovipneumoniae* NXNK2203.

### Genome features

**Fig. 4:**
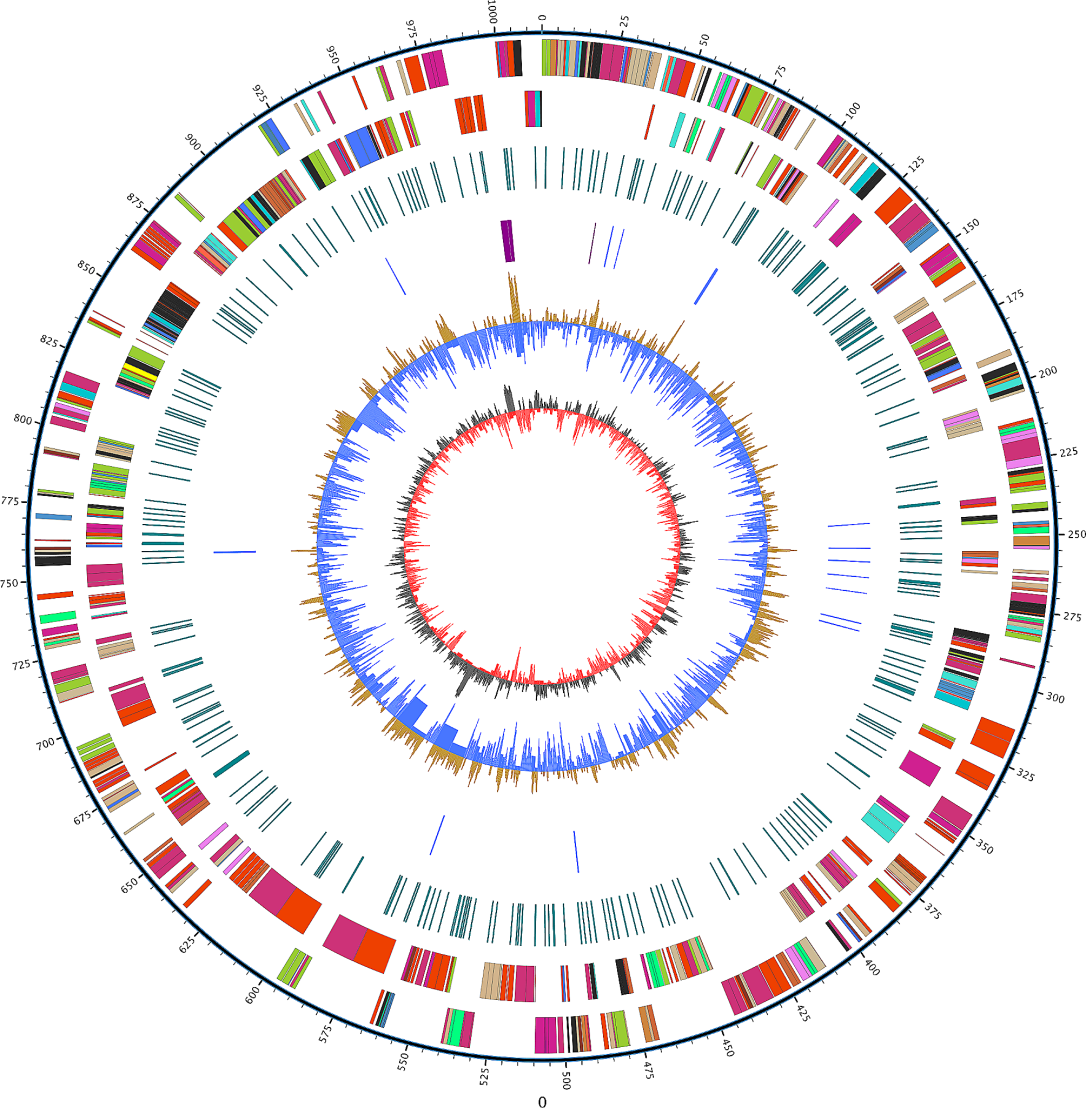
Structure of the *M. ovipneumoniae* NXNK2203 Genome as depicted by a Circos Diagram. The scale is represented by the outermost black circle. Moving inward, the first and second circles depict expected coding sequences on the plus and minus strands, colored according to several functional categories. The third circle depicts the distribution of sequences that repeat. The fourth circle depicts tRNA (blue) and rRNA (red) (purple). The fifth and sixth (innermost) circles reflect the average centered G + C content of the genome (red-above mean, blue-below mean) and the GC skew (GC/(G + C)), respectively

The genomic analysis of the *M. ovipneumoniae* strain NXNK2203 revealed several key features. The strain’s NXNK2203 genome consist of a single circular chromosome of 1,014,835 bp with a GC content of 29.23%. It encodes 3 rRNA and 30 tRNA. A repetitive sequence analysis identified the total length of 12,502 bp, representing 1.23% of the genome (Fig. 4). Furthermore, a genomic island spanning 61,010 bp (559,298 to 620,307) was predicted, containing 13 genes from GE000380 to GE000393. The genome also exhibited the presence of 6 clustered Regularly Interspaced Palindromic Repeats (CRISPR) (additional file 2), 4 prophage elements (additional file 3), and 258 promoter regions (additional file 4).

**Fig. 5:**
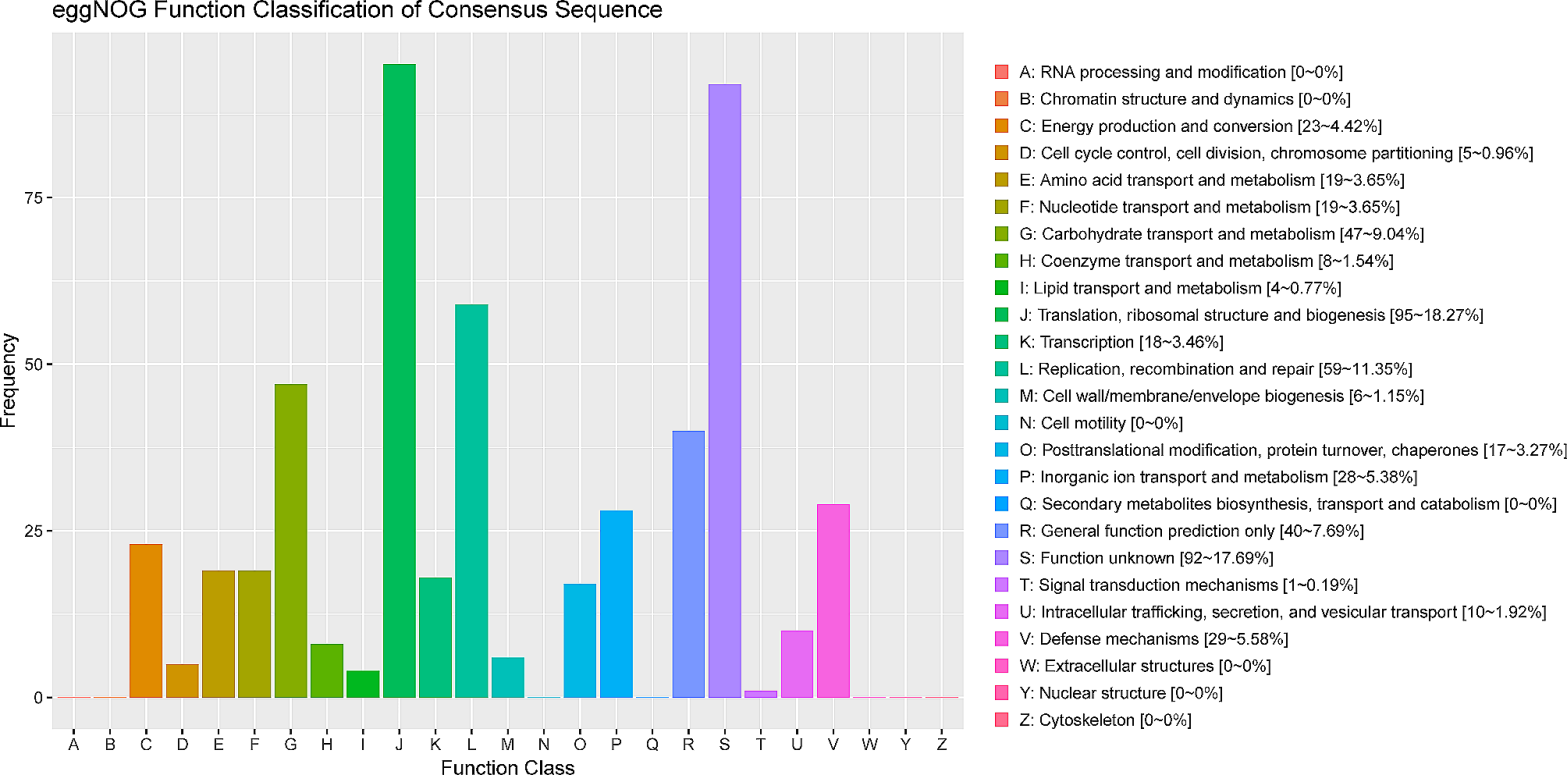
eggnog annotation *M. ovipneumoniae* NXNK2203

The annotation of coding sequences (CDSs) identified 686 genes, which is fewer compared to other strains deposited in the NCBI database (additional file 1). Among these CDSs, 516 (75.2%) genes could be classified into 18 functional categories based on the eggNOG database (Fig. 5 and additional file 5). Additionally, 540 (78.7%) genes could be classified into GO families comprising 3 functional categories (Figure. S1A and additional file 5), while 375 (54.7%) genes could be classified into 3 functional categories according to KEGG database (Figure. S1B and additional file 5). Moreover, 683 genes (99.6%) showed homology to genes within the non-redundant (Nr) database (Figure. S1C and additional file 5), 488 (71.1%) genes could be classified into different families using Pfam database (additional file 5), 390 (56.9%) genes were predicted by Swissprot database (additional file 5), and 683 (99.6%) genes existed in TrEMBL database (additional file 5).

Although no antimicrobial resistance genes were identified, the special database analysis revealed the presence of 73 potential virulence proteins (additional file 6). Among these, several proteins were associated with the Type III, IV, VI, and VII secretion systems (e.g., TTSS, Dot/Icm, ESX-1, HSI-I). The virulence factors like adherence (e.g. FHA and P97/P102 paralog family), Iron uptake (e.g. MgtBC, Enterobactin and FbpABC), Membrane-damaging (e.g. Hemolysin) and proinflammatory effect (e.g. HP-NAP) were also identified. Secreted proteins are considered crucial for pathogenesis, [38] thus signal peptide and transmembrane domain predictions were performed for *M. ovipneumoniae* strain NXNK2203. The analysis identified 31 proteins with secretory signal peptides (additional file 7) and 211 proteins with transmembrane domains (additional file 8). Removal of proteins containing transmembrane helices from the predicted secretory proteins yielded a set of 30 putative secretory proteins (additional file 9). Additionally, the strain exhibited 115 transport proteins involved in the transport of small molecules such as potassium ions (K+), magnesium ions (Mg2+), glucose, and amino acids (additional file 10), highlighting their potential role in bacterial virulence.

### Evolutionary analysis

**Fig. 6:**
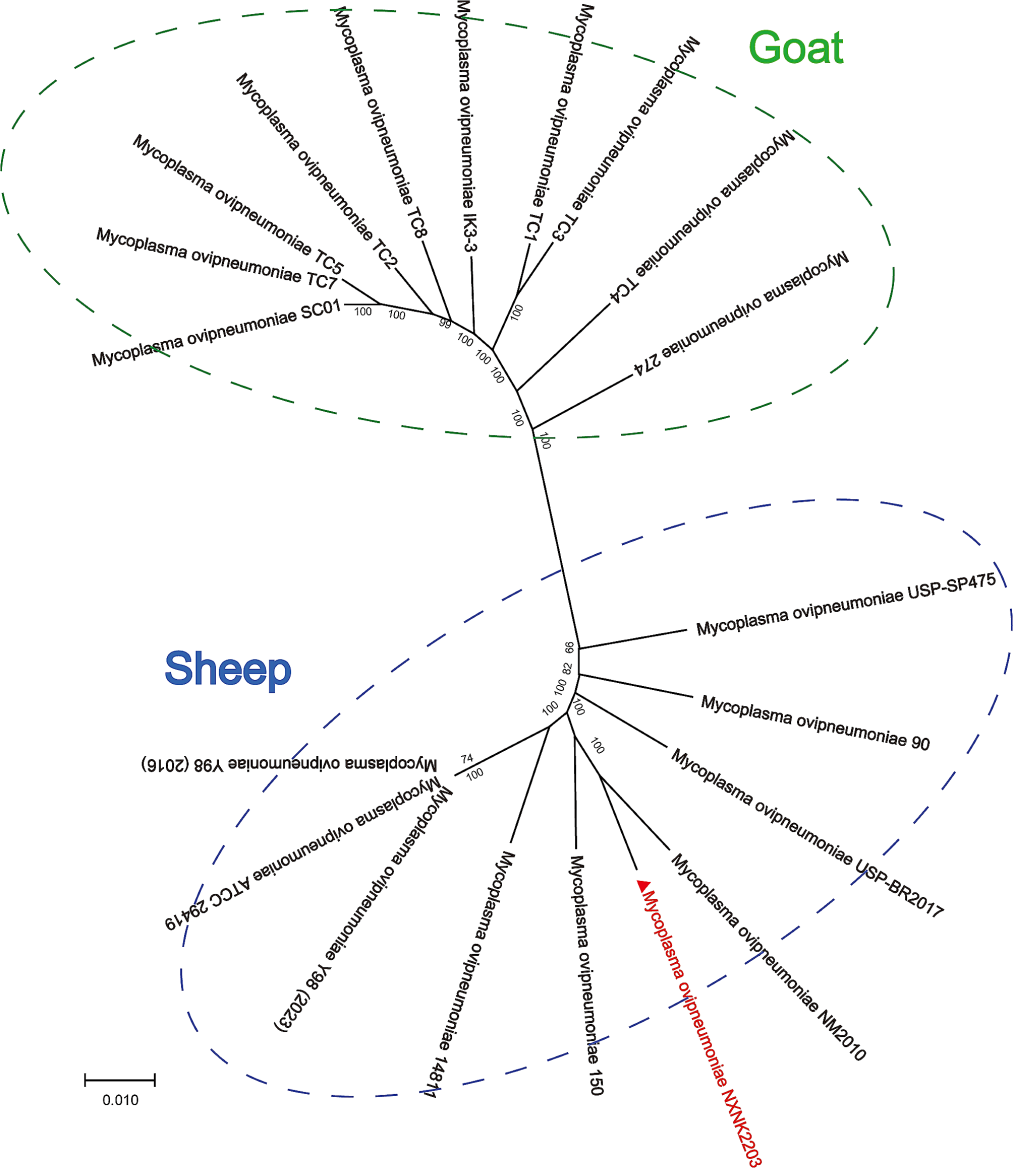
Phylogenetic tree. Phylogenetic relationships of consensus sequences of 19 *M. ovipneumoniae* strains with complete genomic sequences. The phylogenetic groups, goat and sheep as hosts are indicated by green and blue dashed boxes, respectively. Strains marked in red font were isolated in our laboratory

The evolutionary analysis is important in elucidating the genetic relationships and evolutionary history of bacterial pathogens, providing valuable insights into the transmission patterns and host specificity of this pathogen. Evolutionary tree analysis was conducted using the *M. ovipneumoniae* strain NXNK2203 in conjunction with 19 other *M. ovipneumoniae* strains currently deposited in the NCBI database. The resulting phylogenetic tree revealed the presence of two distinct clades within *M. ovipneumoniae*, with separation based on the infected host species (Fig. 6). Specifically, one clade comprised 10 strains associated with goat hosts, including SC01 [15], which represents the first whole genome sequenced in this group. In accordance with expectations, our strain NXNK2203, isolated from Hu sheep, fell within the clade associated with sheep hosts. Furthermore, the evolutionary analysis indicated that *M. ovipneumoniae* strain NXNK2203 exhibited the closest evolutionary relationship with the NM2010 strain (isolated from Inner Mongolia, China) and the 150 strain (isolated from Central Bosnia Canton) [39].

## Discussion

*M. ovipneumoniae* is a prominent pathogen responsible for respiratory infections in both goats and sheep. It is common to observe co-infection of *M. ovipneumoniae* with other microbes, such as *Pasteurella multocida*, *haemolytica*, and the Parainfluenza virus, which exacerbates the severity of the condition. Sheep are particularly susceptible to *M. ovipneumoniae* infection, with a rapid disease progression and more severe consequences compared to goats. However, the susceptibility to *M. ovipneumoniae* infection varies among different sheep breeds. This study aims to explore the vulnerability of Hu sheep to *M. ovipneumoniae* infection and investigate the potential influence of cold weather conditions in late spring on disease progression.

To investigate the susceptibility of Hu sheep to *M. ovipneumoniae* infection, we conducted an autopsy on a severely ill individual from this breed. We hypothesized that the susceptibility might be influenced by the presence of cold weather conditions in late spring. Pathological changes in the sheep’s tissue were carefully examined, and *M. ovipneumoniae* NXNK2203 was isolated and confirmed through laboratory procedures.

The autopsy of the severely affected Hu sheep revealed significant pathological changes in the tissue, providing further evidence of *M. ovipneumoniae* infection. Notably, Hu sheep demonstrated increased susceptibility to *M. ovipneumoniae* infection, which appeared to be further exacerbated by the cold weather conditions prevalent during late spring. These findings align with previous studies highlighting breed-specific susceptibility to *M. ovipneumoniae* infection, with Hu sheep displaying higher vulnerability compared to Duolang and Kazak sheep [40].

The observed higher susceptibility of Hu sheep to *M. ovipneumoniae* infection underscores the importance of considering breed-specific vulnerabilities. Our findings suggest that factors such as genetic predisposition, environmental conditions, and host-pathogen interactions contribute to the varying susceptibility among sheep breeds. Cold weather conditions in late spring emerged as a potential influencing factor, exacerbating the severity of *M. ovipneumoniae* infection in susceptible Hu sheep. Further investigations are warranted to elucidate the mechanisms underlying the interaction between cold weather and *M. ovipneumoniae* infection in sheep.

To gain deeper insights into the pathogenesis of *M. ovipneumoniae*, we conducted whole-genome sequencing of the NXNK2203 strain. Our analysis revealed an intriguing observation regarding the genome size and encoded proteins of *M. ovipneumoniae* in comparison to other strains. Unlike some of the other strains where the genome size is reduced, *M. ovipneumoniae* maintains a comparable genome size. However, there is a reduction in the number of encoded proteins (Table S4). This reduction in encoded proteins could be attributed to the evolutionary process undergone by mycoplasmas. Mycoplasmas have evolved through a degenerative mechanism, which involves the gradual loss of genes that are redundant or nonessential for cell growth. In this process, the genomes of mycoplasmas tend to retain a high proportion of nonredundant genes that are vital for cellular functions [41]. The presence of a reduced number of encoded proteins in *M. ovipneumoniae* may reflect its adaptation to a specialized niche or a host-dependent lifestyle. By streamlining its genetic repertoire, *M. ovipneumoniae* may have optimized its ability to persist and thrive in its specific environment, potentially enhancing its pathogenicity. Further investigations into the specific functions and roles of these proteins are warranted to unravel the underlying mechanisms contributing to the pathogenic nature of *M. ovipneumoniae*. Understanding the genetic elements associated with *M. ovipneumoniae*’s pathogenicity is crucial in unraveling the intricate relationship between this bacterium and the onset of deadly diseases. By characterizing the specific genes and proteins involved in its virulence, antibiotic resistance, and other pathogenic traits, we can gain insights into its mechanisms of infection and develop targeted strategies for diagnosis, treatment, and prevention. It is worth noting that the reduced number of encoded proteins in *M. ovipneumoniae* does not imply a compromised pathogenic potential. In fact, this streamlined genetic makeup may reflect a specialized adaptation to its host and niche environment. The retained nonredundant genes may be essential for crucial processes such as adhesion, invasion, immune evasion, or modulation of the host immune response. Uncovering the functions and mechanisms of these encoded proteins will contribute to a comprehensive understanding of the pathogenesis of *M. ovipneumoniae.*

In addition to the reduced number of encoded proteins, further analysis of the whole genome also revealed the presence of six CRISPR regions in *M. ovipneumoniae*. CRISPR systems are prokaryotic adaptive immune systems that play a crucial role in defending against invading DNA, particularly viruses [42]. The presence of CRISPR regions suggests that *M. ovipneumoniae* has the capacity to mount a defense against foreign genetic material. Interestingly, it has been reported that Mollicutes pathogens, including significant pathogens affecting ruminants, plants, and humans, generally lack the CRISPR/Cas system [43]. However, *M. ovipneumoniae* stands out as one of the few ruminant mycoplasmas possessing a functional CRISPR/Cas system. This unique characteristic makes *M. ovipneumoniae* an intriguing subject for further investigation into the role and potential applications of its endogenous CRISPR/Cas system, including gene knockout experiments [44]. The endogenous CRISPR/Cas system of *M. ovipneumoniae* could potentially serve as a valuable tool for genetic manipulation and gene editing within the bacterium itself. The ability to precisely edit and modify genes in *M. ovipneumoniae* using its own CRISPR/Cas system would greatly advance our understanding of its pathogenicity and open up possibilities for targeted interventions.

The genome annotation of *M. ovipneumoniae* strain NXNK2203 reveals the presence of a substantial number of virulence factors. These factors play crucial roles in various processes, including adhesion, membrane damage, energy transport, and inflammation. These findings align with previous studies that have explored the pathogenic mechanisms of *M. ovipneumoniae* [45]. One of the notable findings in our analysis is the involvement of *M. ovipneumoniae* in glycerol import and the production of reactive oxygen species (ROS). Glycerol import is an essential process for the bacterium, as it serves as a carbon and energy source. The production of ROS, such as hydrogen peroxide (H_2_O_2_), by *M. ovipneumoniae* is significant in terms of its pathogenicity. ROS are highly reactive molecules that can cause oxidative damage to host cells, contributing to the development of inflammatory responses and tissue damage [46].

Moreover, an important virulence component of *M. ovipneumoniae* is the capsule polysaccharide (CPS). CPS contributes to the inflammatory response triggered by *M. ovipneumoniae* infection [47]. It has been observed that CPS can induce the production of ROS, which in turn activates specific signaling pathways in host cells. In particular, CPS-induced ROS production has been found to activate the JNK (c-Jun N-terminal kinase) and p38 mitogen-activated protein kinase (MAPK) signal pathways. These pathways are involved in regulating cellular responses, including inflammation and apoptosis. The activation of JNK and p38 MAPK pathways ultimately leads to the apoptosis of sheep epithelial cells, further highlighting the pathogenic potential of *M. ovipneumoniae*.

Furthermore, in our analysis, we successfully predicted the presence of 30 secreted proteins in *M. ovipneumoniae*. Secreted proteins play a crucial role in the interaction between bacteria and their environment, including host organisms. Previous studies have reported that secreted proteins in Mycoplasma species are released through various mechanisms such as polypeptides, exopolysaccharides, and extracellular vesicles [48]. Despite the identification of these secreted proteins, their specific functions remain largely unknown. However, in a related study, researchers have identified 178 secretory proteins from *Mycoplasma bovis* strain HB0801 [49]. Among them, MbovP0145 [50], MbovP280 [51], and MbovP475 [52] can promote inflammatory response, apoptosis and proliferation, respectively. These findings demonstrate the potential significance of secreted proteins in modulating cellular processes and host-pathogen interactions.

In the case of *M. ovipneumoniae*, little research on the secreted protein has conducted. Therefore, further exploration and comprehensive analysis are required to gain a better understanding of the secreted proteins and their roles in the virulence of *M. ovipneumoniae*. By investigating the functions and mechanisms of these secreted proteins, we can potentially uncover critical virulence factors and enhance our knowledge of the pathogenicity of *M. ovipneumoniae*. This information will contribute to the development of targeted therapeutic interventions and strategies to combat the deadly diseases caused by this bacterium.

## Conclusion

This study highlights the heightened vulnerability of Hu sheep to *M. ovipneumoniae* infection, particularly in the context of cold weather during late spring. The detailed autopsy and examination of pathological changes provide a better understanding of the disease progression in Hu sheep. The successful isolation of *M. ovipneumoniae* NXNK2203 and the subsequent whole genome sequencing analysis contribute to the identification and characterization of genetic elements related to pathogenicity, and virulence factors of the bacteria. These findings have implications for the management and control of *M. ovipneumoniae* infections in Hu sheep, aiding in the development of targeted interventions and preventive strategies to minimize the impact of this pathogen on sheep health and welfare.

### Electronic supplementary material

Below is the link to the electronic supplementary material.

Supplementary Material 1

Supplementary Material 2

Supplementary Material 3

Supplementary Material 4

Supplementary Material 5

Supplementary Material 6

Supplementary Material 7

Supplementary Material 8

Supplementary Material 9

Supplementary Material 10

Supplementary Material 11

Supplementary Material 12

## Data Availability

The datasets presented in this study can be found in online repositories. The complete *M. ovipneumoniae* strain NXNK2203 genome sequence was deposited at NCBI GenBank (Accession number: CP124621).
