# Supplementary figures and images for: Exploring Mycoplasma ovipneumoniae NXNK2203 infection in sheep: insights from histopathology and whole genome sequencing
Source: BMC Vet Res. 2024 Jan 10;20:20. doi: 10.1186/s12917-023-03866-z (PMC10777581; doi:10.1186/s12917-023-03866-z)

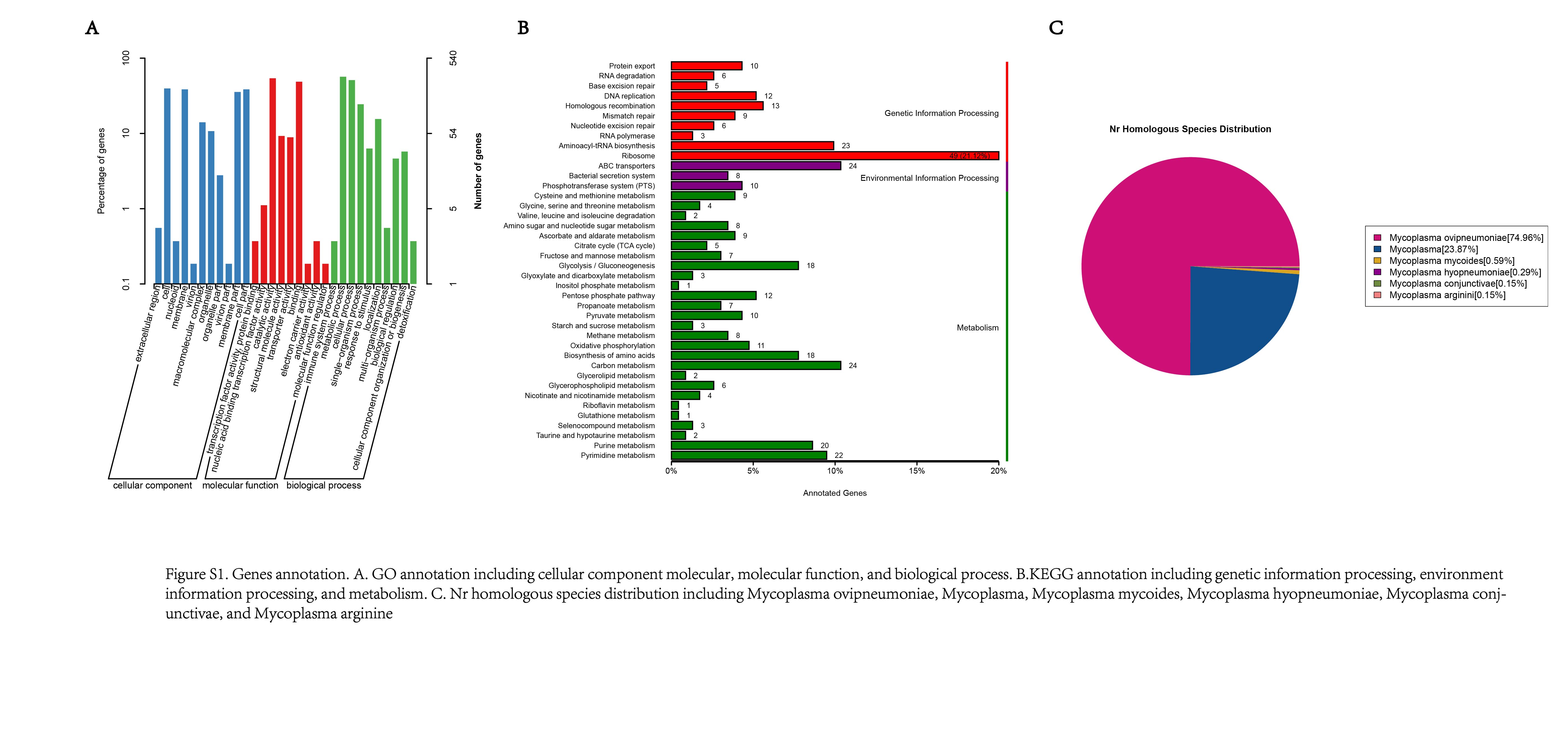

Supplement: Supplementary file 11 — Supplementary Material 11 [file 12917_2023_3866_MOESM11_ESM.jpg]

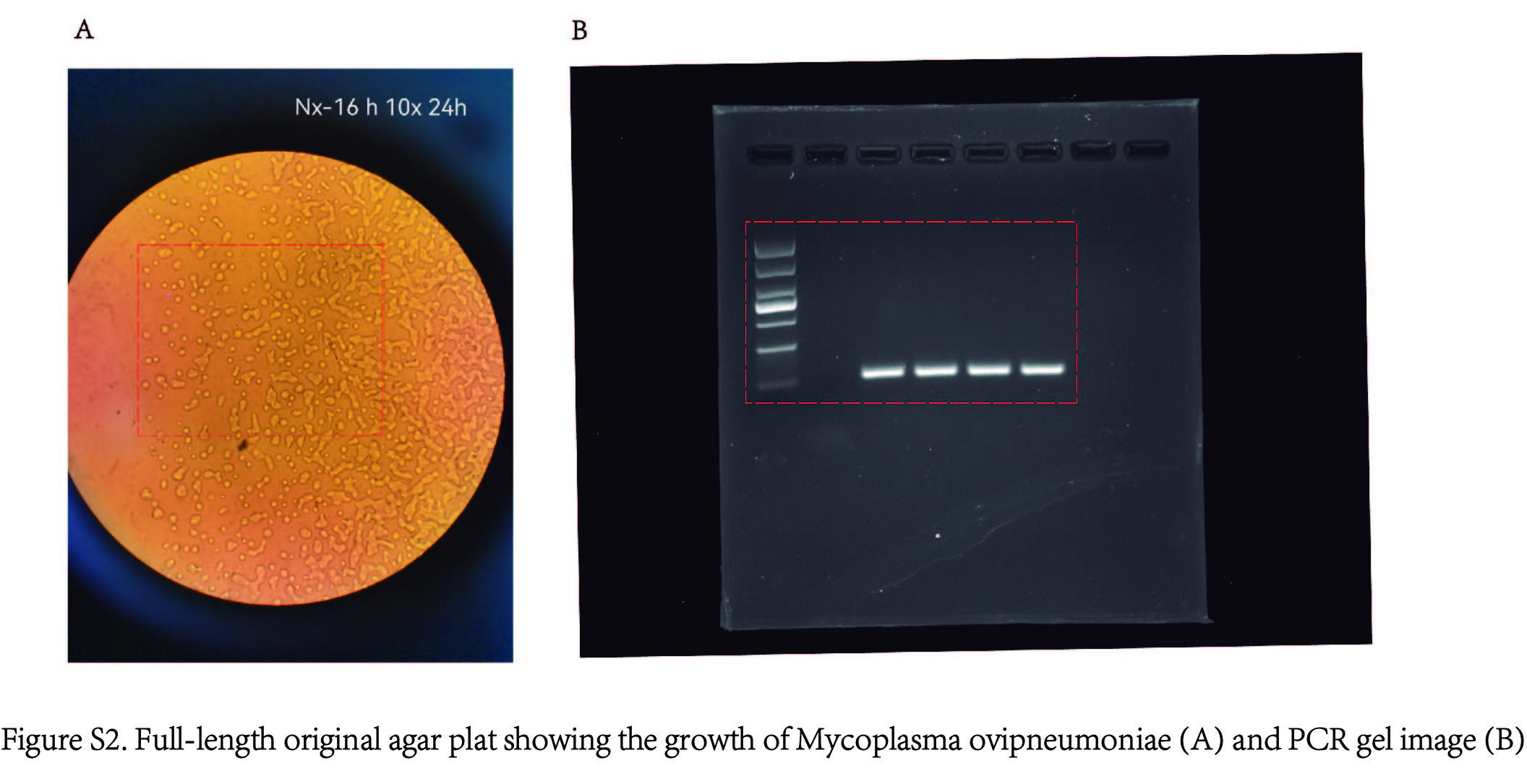

Supplement: Supplementary file 12 — Supplementary Material 12 [file 12917_2023_3866_MOESM12_ESM.jpg]
